# Evolution of the prostate cancer diagnostic paradigm: integrated evidence and clinical pathways from targeted biopsy to biopsy-free strategy

**DOI:** 10.3389/fonc.2026.1835580

**Published:** 2026-06-24

**Authors:** Zhiyong Liu, Jianhe Wu, Qiang Lu, Yuanwei Li, Yongjun Yang

**Affiliations:** 1 Department of Urology, Hunan Provincial People’s Hospital, The First Affiliated Hospital of Hunan Normal University, Changsha, Hunan, China; 2 Clinical Medical College, Hunan Normal University, Changsha, Hunan, China

**Keywords:** biopsy, diagnosis, multiparametric magnetic resonance imaging, prostate cancer, prostate-specific antigen, PSMA-PET/CT

## Abstract

The field of prostate cancer (PCa) diagnosis is undergoing a profound transformation from traditional invasive procedures toward precision, minimally invasive, and even non-invasive approaches. This article systematically outlines this evolutionary trajectory: first, starting with the selection of patients for biopsy, it emphasizes precision-based initial screening and risk-stratified biopsy strategies utilizing multiparametric magnetic resonance imaging (mpMRI) and prostate-specific antigen density; second, by comparing transrectal and transperineal biopsy, it elucidates the significant advantages of the transperineal approach in reducing the risk of infection and its status as the new standard pathway; further, it focuses on how biopsy strategies have evolved from systematic template biopsy to targeted, individualized fusion biopsyunder the guidance of precision imaging modalities such as mpMRI and prostate-specific membrane antigen positron emission tomography/computed tomography; finally, we explore the application prospects, clinical validation pathways, and practical frameworks of the new “biopsy-free diagnosis” paradigm—based on advanced imaging and molecular biomarkers—in radical surgery decision-making and active surveillance. The full text integrates the latest evidence-based data and proposes a structured clinical pathway, aiming to advance PCa diagnosis into a new era characterized by greater safety, precision, and individualization.

## 1 Introduction

Prostate cancer (PCa) is a common malignancy that seriously threatens the health of middle-aged and elderly men. Traditional diagnosis primarily relies on serum prostate-specific antigen (PSA) screening, with those showing abnormal results often requiring transrectal ultrasound-guided (TRUS) biopsy. However, this invasive procedure not only carries risks such as bleeding and infection, but its random sampling nature also frequently leads to the missed diagnosis of clinically significant prostate cancer (csPCa) or the overdiagnosis of clinically insignificant prostate cancer (cisPCa). Against this backdrop, this article aims to systematically outline the three core components of this evolutionary pathway: first, to explore how to achieve precise selection of biopsy candidates using tools such as multiparametric magnetic resonance imaging (mpMRI) and prostate-specific antigen density (PSAD); second, to elucidate the paradigm shift in biopsy safety by comparing transrectal and transperineal biopsy; finally, to analyze the strategy evolution from systematic biopsy to targeted and personalized biopsy, and on this basis, to outline the clinical validation pathway for the new paradigm of “biopsy-free diagnosis.” By integrating the latest evidence-based data, this paper proposes a structured clinical pathway to serve as a reference for PCa diagnosis toward safer, more precise, and more personalized approaches.

## 2 Patient selection for prostate biopsy: from broad screening to precision screening and risk stratification biopsy

The diagnostic value of prostate biopsy is undisputed; however, as an invasive procedure, it carries risks of bleeding, infection, and even more severe complications. Therefore, a core shift in contemporary clinical practice lies in moving from universal biopsy of “suspected cases” to “precision screening” and “risk-stratified biopsy” based on multidimensional evidence. This shift aims to maximize the detection of csPCa while minimizing overdiagnosis and harm to patients with indolent disease.

Traditionally, elevated PSA levels or an abnormal digital rectal exam have been the primary indications for biopsy. However, the low specificity of PSA leads to a large number of unnecessary biopsies and the detection of cisPCa. Advances in imaging, represented by mpMRI, have completely transformed this landscape. The landmark PRECISION trial demonstrated that, for patients with elevated PSA, a strategy of performing mpMRI first and conducting targeted biopsy only when suspicious lesions (Prostate Imaging Reporting and Data System (PI-RADS) ≥ 3 are identified increases the detection rate of csPCa by 12% (38% vs. 26%) compared to standard TRUS-guided biopsy, while reducing unnecessary biopsies by 28% (1). This establishes the “gatekeeper” role of mpMRI in the diagnostic workflow. Subsequent real-world studies, such as the 4M trial, have further validated the effectiveness of this strategy (2). Consequently, the core indication for the initial biopsy has been refined to “clear suspicious lesions identified by mpMRI.” For patients with mpMRI-negative results (PI-RADS 1–2), particularly those with low PSAD, the risk of missing high-grade cancer is extremely low (<3%) (3, 4). For such patients, active surveillance (AS) rather than immediate biopsy has become the standard approach, significantly reducing the clinical burden and patient anxiety (5).

Current risk stratification has evolved from relying on a single imaging modality to a multimodal precision assessment system that integrates clinical, molecular, and algorithmic models, aiming to provide quantitative decision-making support for different clinical scenarios.

Regarding the diagnostic challenge of PI-RADS 3 lesions on mpMRI, a multicenter study by Mjaess et al. effectively distinguished the benign and malignant risks within this group by establishing more refined PSAD cutoffs, providing clear biopsy guidance for this large population with diagnostic uncertainty (6). Concurrently, a study by Ji et al. demonstrated that optimized combination models of traditional clinical parameters can achieve effective risk stratification even without relying on advanced imaging, providing a reliable tool for resource-limited settings (7).

Risk stratification is being deeply expanded into the realm of molecular biology. Research by Olah et al. confirms that novel tissue biomarkers can significantly enhance prognostic stratification for patients with localized PCa, identifying high-risk subgroups beyond those identified by traditional pathology (8). Methodologically, the machine learning models and online calculators developed by Vasconcelos Ordones et al. integrate multidimensional data and capture complex nonlinear relationships, providing predictive tools with superior performance and clinical usability (9).

For traditionally defined high-risk patients, their internal heterogeneity also needs to be identified. Through a meta-analysis of multiple randomized trials, Ravi et al. achieved refined subgrouping of high-risk/locally advanced PCa, which will help ensure precise matching of treatment intensity in the future and avoid overtreatment or undertreatment (10).

From single-modality imaging to multi-parameter integration, from morphological assessment to exploration of biological significance, and from general categorization to precise segmentation tailored to specific clinical contexts—future stratification systems will be dynamic, intelligent, and integrated throughout the entire diagnostic and therapeutic process.

Currently, for patients with clinically high-suspicion PCa who have a negative initial biopsy, the core of the repeat biopsy strategy has shifted to “targeted sampling of well-defined lesions under precise image guidance”. MpMRI is the cornerstone of this strategy. Systematic reviews have shown that for men with mpMRI-positive findings and a negative initial biopsy, repeat targeted biopsy of imaging-suspicious lesions achieves a csPCa detection rate of 24%–52%, establishing the role of imaging as the primary basis for repeat biopsy decisions (11). In practice, mpMRI/TRUS-guided targeted biopsy technique is significantly superior to conventional systematic biopsy, particularly in effectively detecting tumors in the anterior prostate that are easily missed by routine needle biopsy (12).

Barone et al. further focused on patients undergoing repeat biopsy who had previously tested negative but exhibited persistently elevated PSA levels. They found that the performance of mpMRI in detecting csPCa (defined as a Gleason score ≥3 + 4 or a tumor length ≥6 mm) was comparable to that in the initial diagnosis population (39.0% vs. 36.1% for PI-RADS 4) (13). Compared to the systematic review by Grivas et al., this study employed a stricter definition of csPCa and a slightly higher mean patient age (68 years), which may have resulted in a slightly lower detection rate, suggesting that differences in inclusion/exclusion criteria should be considered when interpreting the conclusions of different studies (11, 13).

Evidence suggests that for patients with clearly suspicious lesions on mpMRI, if high-quality targeted biopsy results are negative, the incremental value of additional systematic biopsy is limited; conversely, for those with unclear targets or poor biopsy conditions, combined systematic biopsy remains necessary (14). In addressing the clinical dilemma of “persistently positive imaging but negative biopsy”, PSAD serves as a key stratification tool. For patients with a PSAD ≥0.15 ng/ml/cc, the risk of detecting csPCa on repeat biopsy is significantly increased, and repeat targeted biopsy should be actively considered; conversely, for those with a lower PSAD, close imaging surveillance may be selected (15). For patients with PI-RADS 4/5 lesions but negative fusion-guided biopsy results, short-term follow-up data support AS rather than immediate repeat invasive procedures, reflecting the prudent principle of avoiding overtreatment (16).

When there is a discrepancy between mpMRI and clinical judgment, prostate-specific membrane antigen positron emission tomography/computed tomography (PSMA-PET/CT) provides a higher level of guidance. Prospective studies have confirmed that, in such challenging cases, PSMA-PET/CT-guided biopsy outperforms mpMRI in detecting csPCa, offering a more precise solution for repeat biopsies (17). It is worth noting that even when the initial biopsy was performed under mpMRI guidance, repeat evaluation remains valuable for patients at persistently high risk, with detection rates still reaching substantial levels, underscoring the importance of comprehensive management based on dynamic risk assessment (18).

In summary, repeat biopsy has evolved from blind sampling into a precision process centered on mpMRI, utilizing risk stratification tools such as PSAD, and guided by PSMA-PET/CT when necessary. Its essence lies in precisely targeting well-defined imaging lesions while identifying patients who can safely benefit from AS through risk stratification, ultimately achieving an optimal balance between diagnostic and therapeutic efficiency and patient safety.

## 3 Selection of prostate biopsy approaches: an in-depth evidence-based analysis and paradigm shift of transrectal and transperineal routes

Having established the principles of precise initial screening based on mpMRI and PSAD, the safety of the biopsy procedure itself becomes the next critical step. The choice of biopsy approach directly determines the risk of infection and is a core step in shifting the diagnostic paradigm toward “greater safety.”

The diagnostic paradigm of prostate biopsy is undergoing a profound revolution in access routes, driven by the pursuit of patient safety and precision medicine. The traditional landscape, dominated by the transrectal approach, has been completely reshaped by high-quality clinical evidence. Based on a series of landmark studies from the past five years—including large-scale randomized controlled trials (RCTs), prospective systematic reviews, and economic analyses—this section provides an in-depth analysis of the overall performance of the transrectal and transperineal approaches, elucidating the core logic that clinical decision-making should shift from prioritizing “procedural convenience” to prioritizing “patient safety.”

### 3.1 Transrectal prostate biopsy: reassessment and repositioning of the traditional approach

Transrectal prostate biopsy (TR-PB) has been in use for decades due to its technical maturity, lack of requirement for specialized equipment, and ability to be rapidly performed on an outpatient basis under local anesthesia. However, its fundamental flaw—the need for the biopsy needle to penetrate the rectal mucosa, which is colonized with opportunistic pathogens—poses an unavoidable risk of infection.

Recent studies have confirmed that the risk of infection with TR-PB remains significant even when antibiotic prophylaxis guidelines are followed. The PREVENT trial, a multicenter RCT, provides the most direct comparison: in the context of mpMRI/TRUS-guided biopsy, the incidence of infection-related complications in the TR-PB group reached 7.0%, whereas it was only 0.5% in the TP-PB group (19). The TRANSLATE trial, the largest relevant RCT to date, further reported that the incidence of confirmed sepsis in the TR-PB group was 2.1%, significantly higher than the 0.1% in the TP-PB group (20). These data indicate that the risk of infection is not a low-probability event but rather a significant clinical issue affecting nearly one in ten patients. A network meta-analysis focused on infection prevention also concluded that TR-PB with empirical antibiotic prophylaxis remains one of the strategies with the highest risk of infection compared to TP-PB (21).

In terms of diagnostic performance, TR-PB has not been completely ruled out. The TRANSLATE trial confirmed that for the detection of csPCa, TR-BP (detection rate of 30%) achieved non-inferiority to TP-PB under local anesthesia (detection rate of 29%) (20). Several systematic reviews and meta-analyses have also indicated that there is no statistically significant difference between the two approaches in terms of overall csPCa detection rates (22–25). However, it is worth noting that an earlier but frequently cited meta-analysis suggested that the TP-PB approach may offer an advantage in detecting csPCa during mrMRI/TRUS-guided biopsy, particularly for anterior lesions (26). A more recent systematic review by Zattoni et al. of prospective studies showed that, under strictly matched mpMRI/TRUS-guided conditions, the detection rates of the two approaches were comparable, but the TP-PB group demonstrated superior safety (22).

Given its well-defined risks, the role of TR-PB should be strictly limited. It may only be considered as an alternative option in healthcare facilities where TR-PB is not yet widely available, for patients with an extremely low risk of infection (no colonization by drug-resistant bacteria, no history of recurrent urinary tract infections, and normal immune function) and whose prostate anatomy (e.g., lesions clearly located in the posterior zone) is suitable. Any decision must be based on fully informed consent, with clear disclosure of the higher risk of infection (27, 28).

### 3.2 Transperineal prostate biopsy: establishment and optimization of the new standard protocol

The transperineal approach fundamentally reduces the risk of infection by bypassing the rectum and, with technological advances, has enabled outpatient procedures and greater precision.

The PREVENT and TRANSLATE trials provide the strongest RCT evidence regarding the safety of TP-PB, demonstrating extremely low rates of infection-related complications and sepsis (19, 20). Several other meta-analyses have consistently confirmed this advantage (21, 23, 24, 29). The clinical significance of this shift in safety is revolutionary: it transforms prostate biopsy from an invasive procedure requiring “antibiotic escort” into a safe procedure centered on local aseptic technique, similar to many other minor outpatient surgeries.

TP-PB does not sacrifice diagnostic accuracy for safety. The TRANSLATE trial (20) and the systematic review by Zattoni et al. (22) have both confirmed its non-inferiority in diagnostic performance. Furthermore, because the needle enters through the perineum, it can more easily reach the anterior and apical regions of the prostate at an anterior-tilted angle, which anatomically facilitates precise targeting of mpMRI-suspicious lesions in these areas. A systematic review specifically comparing the two approaches under mpMRI/TURS fusion guidance noted that the TP-PB approach may have theoretical and technical advantages for sampling cancer in the anterior prostate (30).

Early TP-PB procedures often required regional or general anesthesia in the operating room, limiting their widespread adoption. However, recent practice has completely transformed this situation. Studies by Hong et al. confirmed that outpatient TP-PB under local anesthesia is safe, feasible, and well-tolerated (31). Bryant et al. employed a local anesthesia protocol in the TRANSLATE trial, validating its feasibility in large-scale, multicenter practice (20). Learning curve studies indicate that physicians can become proficient in the outpatient free-hand cognitive fusion TP-PB technique after performing approximately 20–30 procedures (32). A subanalysis of patient-reported outcomes from the ProBE-PC trial further demonstrated that patients’ experiences of pain and anxiety during TP-PB under local anesthesia did not differ significantly from those during TR-PB, but the former group expressed fewer concerns about complications (33).

Although the per-procedure cost of TP-PB may be slightly higher due to equipment or a slightly longer operative time, the substantial reduction in infection-related expenditures confers a health economic advantage. An economic analysis by Huang et al. based on data from the PREVENT trial showed that, from a payer’s perspective, mpMRI/TRUS-guided TP-PB performed in an office setting is cost-effective compared to TR-PB (34).

### 3.3 Comprehensive comparison: from multidimensional evidence to clinical decision-making

The TRANSLATE and PREVENT trials are the two cornerstone studies underpinning current decision-making (see **Table 1**).

**Table 1:** Comprehensive comparison of TR-PB and TP-BP.

Comparison dimensions	Transrectal biopsy (TR-PB)	Transperineal biopsy (TP-PB)	Key evidence-based findings and interpretation
Core safety: infection	High risk. The incidence of sepsis can reach 1–3%, even with targeted antibiotic prophylaxis	Very low risk. The incidence of sepsis is <0.5%, and oral antibiotic prophylaxis is generally not required	Evidence Grade: High (multiple RCTs). Differences in infection risk are the primary driver of the paradigm shift. The PREVENT and TRANSLATE trials provide decisive evidence
Diagnostic performance (Detection of csPCa)	Effective, but limited by anatomy. Overall detection rates are comparable to TP-PB, but targeted biopsy of anterior lesions may be limited	Non-inferior, with anatomical advantages. Overall detection rate is non-inferior to TR-PB; sampling of anterior lesions is more direct, potentially improving targeting accuracy	Level of evidence: High (large RCT). The TRANSLATE trial established non-inferiority in diagnostic performance. Systematic reviews also support this conclusion
Patient-reported outcomes	Procedure-related pain is manageable, but patients report high levels of anxiety regarding infection	Pain and anxiety levels under local anesthesia are comparable to those of TR-PB; however, due to the lower risk of infection, overall satisfaction and sense of safety are higher	Evidence grade: Moderate-high (RCT subanalysis/cohort studies). Patient-reported outcomes from the ProBE-PC trial provide direct comparative data
Spectrum of non-infectious complications	More common: hematospermia, rectal bleeding, hematuria	More common: transient perineal hematoma, urinary retention. Rare: rectal injury	Evidence grade: High (RCT). The ProBE-PC and TRANSLAT trials provide detailed descriptions and comparisons of the different complication profiles
Health economics	The cost of a single procedure is low, but the cost of managing infectious events is high, potentially increasing the overall healthcare burden	The cost per procedure may be slightly higher, but it is cost-effective in the long term due to the prevention of the vast majority of infections.	Level of evidence: Moderate (model analysis based on RCTs). The economic analysis of the PREVENT trial supports this
Technology and training	The technique is well-established, with a short learning curve and high adoption rates	A learning curve exists but is surmountable. Proficiency in outpatient local anesthesia freehand technique can be achieved after approximately 20–30 procedures. Training is key to widespread adoption	Level of evidence: Moderate (prospective cohort study). Dedicated learning curve studies provide a basis for training planning

A comprehensive comparison of the two approaches is summarized in **Table 1**.

#### 3.3.1 TRANSLATE trial

The TRANSLATE trial (20, 35) was a large-scale RCT conducted in the UK, involving approximately 2,500 patients, addressed two core questions through its rigorous design: 1) In terms of detection rates for csPCa, TP-PB under local anesthesia was non-inferior to TR-PB; 2) Regarding the primary safety endpoint (sepsis), TP-PB was significantly superior to TR-PB. This study directly demonstrated the feasibility and superiority of replacing TR-PB with TP-PB under local anesthesia in routine diagnostic settings.

#### 3.3.2 The PREVENT trial

The PREVENT trial (19) was a U.S.-based RCT focused on evaluating the efficacy of TP-PB in preventing infection-related complications within a combined mpMRI/TRUS-targeted and systematic biopsy approach. The results demonstrated that TP-PB reduced infection-related complications by more than 90%, and a subsequent economic analysis (34) confirmed that this safety advantage translates into economic value.

Based on the above evidence, the selection of the approach for prostate needle biopsy should follow the following clear pathway:

1. Establish perineal biopsy as the preferred and recommended standard approach. For the vast majority of patients undergoing their first or repeat biopsy, TP-PB should be prioritized, and patients should be clearly informed of its significant safety advantages.
2. Transrectal biopsy should be strictly limited to specific scenarios: it may only be considered when the healthcare facility lacks the technical capabilities for TP-PB, and only if the patient’s risk of infection is extremely low and they have provided informed consent to assume the higher risk.
3. Vigorously promote the use of TP-PB under local anesthesia in outpatient settings. Healthcare facilities should allocate resources for physician training (32, 36), establish standardized operating procedures to overcome the initial learning curve, and ensure the widespread adoption of this safer technique (31).
4. Decision-making should be individualized. For patients with mpMRI showing lesions clearly located in the anterior region, or those with high-risk factors for infection, TP-PB is the undisputed best choice (28, 37).

The field of prostate biopsy is undergoing a clear paradigm shift. With patient safety as the central priority, the transperineal approach has accumulated irrefutable evidence-based medical data, demonstrated by its exceptional safety (over 90% reduction in infection risk) and confirmed non-inferior diagnostic capability in major RCTs such as TRANSLATE and PREVENT. It is no longer merely an “alternative” but represents a new standard technique that is safer and more aligned with the principles of modern precision medicine. Future clinical practice and guideline updates should actively embrace this shift, promote the widespread implementation of TP-PB, and ultimately enable more patients to benefit from this safer diagnostic technology.

## 4 The evolution of biopsy strategy: paradigm shifts and personalized practice driven by precision imaging

Having addressed the issues of “who to biopsy” and “how to perform biopsies safely,” the focus has shifted to how to achieve “more precise biopsies” using precision imaging. Biopsy strategy is undergoing a profound transformation from random sampling to individualized navigation.

Core advancements in PCa diagnosis stem from the maturation and integration of precision imaging technologies, exemplified by mpMRI and PSMA-PET/CT. These technologies have not only revolutionized pre-biopsy lesion localization and risk stratification but have also directly driven a profound evolution in biopsy strategies—from “blind randomization” to “image-guided” and ultimately to “multimodal individualized” approaches. This chapter will systematically outline this evolutionary path: first, it will analyze how precision imaging technologies have reshaped the diagnostic paradigm; subsequently, it will discuss how, against this backdrop, biopsy strategies have evolved from systematic template biopsy to targeted biopsy, and are now advancing toward more refined, individualized integrated approaches.

### 4.1 Establishing the paradigm of precision imaging: the evolving roles of mpMRI and PSMA-PET/CT

MpMRI has clearly emerged as a key “gatekeeper” in the PCa diagnostic workflow. Its core value lies in optimizing biopsy decisions through precise triage, ensuring the detection of csPCa while significantly reducing unnecessary biopsies. This role is primarily supported by the following evidence: large randomized trials have confirmed that mpMRI-guided targeted strategies can help approximately 28% of patients avoid immediate biopsy, with diagnostic performance non-inferior to systematic biopsy (38); its high negative predictive value (89–91%) ensures the safety of mpMRI-negative patients entering AS (39); for mpMRI-positive patients, targeted or targeted-plus-regional biopsy can achieve detection rates comparable to those of systematic biopsy with fewer needle insertions (40); a meta-analysis further demonstrates that this strategy can reduce unnecessary biopsies by approximately 40% and increase the detection rate of csPCa by approximately 15% (41); prospective real-world studies have also validated the effectiveness and robustness of this strategy in routine clinical practice (42). Therefore, through reliable risk stratification and lesion localization, mpMRI achieves an optimal balance between diagnostic efficiency and patient safety.

PSMA-PET/CT has evolved from a staging tool into an “advanced navigation” system within the diagnostic workflow, providing critical complementary molecular functional information to mpMRI. Its core value lies in precisely guiding the management of challenging cases and directly influencing treatment decisions.

For cases where mpMRI results are uncertain or inconsistent with clinical judgment, PSMA-PET/CT demonstrates superior navigational efficacy. Prospective studies indicate that, in such situations, PSMA-PET/CT-guided biopsy outperforms mpMRI in detecting csPCa and can serve as an effective arbitration tool (17). Meta-analyses indicate that while its overall performance in local staging of the primary tumor is comparable to that of mpMRI, it exhibits higher specificity in assessing extracapsular extension (ECE) (43); head-to-head studies also confirm that Ga-PSMA PET/CT outperforms conventional mpMRI in localizing the primary lesion and assessing seminal vesicle invasion (44).

As the recommended method for initial staging of intermediate-and high-risk PCa, PSMA-PET/CT can more accurately detect lymph node and distant micrometastases, providing a comprehensive map of the systemic disease (45). Its navigational value directly translates into optimized treatment decisions: prospective trials have shown that, based on PSMA-PET/CT staging and targeted biopsy, more than 40% of patients had their initial treatment regimens altered, achieving precise navigation from imaging to treatment (46).

When mpMRI is not readily available, PSMA-PET/CT can serve as an independent primary imaging modality, guiding targeted biopsy by identifying highly metabolizing lesions and opening a new pathway for precision diagnosis independent of MRI (47).

In summary, through the precise complementarity of local and systemic staging, as well as direct intervention in treatment strategies, PSMA-PET/CT has established itself as a core advanced navigation tool driving the advancement of PCa diagnosis and treatment toward an era of personalized precision medicine. Although PSMA-PET/CT demonstrates significant advantages in precision navigation, its clinical implementation still faces several practical challenges. First, the high costs of equipment and examinations, coupled with the production and distribution constraints of radiotracers (such as ^68^Ga-PSMA-11) due to their half-lives, have prevented the widespread adoption of this technology in most medical centers. Second, PSMA is not a prostate cancer-specific marker; false-positive uptake can occur in conditions such as prostatitis, benign prostatic hyperplasia, and even non-prostatic lesions (e.g., ganglions, hemangiomas), which may misdirect biopsy targeting or lead to unnecessary over-staging. Furthermore, the interpretation of PSMA-PET/CT exhibits inter-observer variability, particularly regarding the determination of SUVmax cutoff values and the identification of micrometastases; standardized training and quality control still need to be strengthened. Therefore, in clinical practice, its added value should be weighed against its limitations to avoid expanding indications in the absence of sufficient evidence.

### 4.2 Synergistic evolution of biopsy strategies: from systemic to targeted, toward personalization

Guided by precision imaging, the technical strategies of needle biopsy itself have also undergone a synergistic evolution, with the goal of achieving an optimal balance between diagnostic efficiency and safety.

In the era of precision imaging, systematic template biopsy has shifted from being the “gold standard” for diagnosis to serving as a limited supplement to targeted biopsy. This shift in role is supported by a clear chain of evidence: first, mpMRI risk stratification can reduce unnecessary biopsies at the source and optimize screening pathways (48); second, fusion-guided targeted biopsy has demonstrated superior efficacy in detecting csPCa (49). When used in combination, systematic biopsy offers limited additional detection of significant cancer, resulting in diminished incremental value (50); whereas the more precise “targeted + ipsilateral systematic” approach reduces invasiveness while maintaining detection rates (51). Health economic analyses also support the cost-effectiveness of the mpMRI-guided approach (52). Therefore, systematic biopsy should currently be strictly reserved for specific scenarios, such as when the targeted approach is negative but the patient remains clinically at high risk; its core role is to provide precise supplementary information with minimal invasiveness to avoid potential limited false negatives.

Image-guided targeted biopsy has been established as the new standard for the precise diagnosis of PCa. The core of this strategy lies in performing high-precision sampling directly on suspicious lesions identified by mpMRI, achieving a paradigm shift from “random screening” to “precision targeting” to prioritize the detection of csPCa with fewer needle samples.

Its status as the standard of care is supported by multiple lines of evidence: First, in screened populations, the cancer detection efficiency per needle in targeted biopsy is significantly higher than that of systematic biopsy, optimizing diagnostic efficiency (53); second, for strictly selected mpMRI-positive patients, targeted biopsy alone is sufficient to detect the vast majority of csPCa, confirming its reliability as an independent diagnostic strategy (54); furthermore, real-world comparative studies have demonstrated that, under mpMRI guidance, the detection rate of csPCa using cognitive fusion-guided targeted biopsy is significantly superior to that of conventional systematic biopsy, highlighting its central role (55); finally, when faced with challenging scenarios such as small lesions or large-volume prostates, both software fusion and cognitive fusion technologies can accurately sample occult lesions, ensuring the precision and universality of the diagnosis (56). Therefore, targeted biopsy is not merely a technical choice but represents a fundamental shift in diagnostic objectives—from “detecting any cancer” to “precisely identifying cancers requiring intervention.”

The core of an individualized biopsy strategy lies in prudently expanding the sampling scope based on “targeted biopsy,” guided by imaging features and clinical risk, to balance the risk of missed diagnosis with procedural trauma. Its primary implementation follows two pathways:

In high-risk scenarios where missed diagnoses must be avoided, adding a systematic biopsy to a targeted biopsy is the standard strategy. Meta-analyses confirm that combined biopsy can further increase the detection rate of csPCa by 5–10%, with the primary findings being csPCa located outside the targeted lesions (57). A retrospective study by Massanova et al. further found that the benefits of this combined strategy exhibit regional heterogeneity: for lesions located in the apex and anterior regions of the prostate, the detection rate of targeted biopsies was significantly higher than that of systematic biopsies, suggesting that the anatomical location of lesions must be fully considered when developing individualized biopsy plans (58). A recent prospective randomized trial by Liu et al. further demonstrated that the combination of targeted and systematic biopsy significantly improved the detection rate of csPCa compared with systematic biopsy alone, with greater benefits observed in patients with PSAD >0.15 ng/mL or PI-RADS 4-5 lesions (59).

For a single high-risk lesion, increasing the sampling density within the target area (saturation biopsy) allows for a more accurate assessment of its true risk. Prospective trials have shown that performing a 9-needle saturation biopsy on the same mpMRI lesion, compared to a standard 4-needle targeted biopsy, increases the pathological upgrade rate by 15% and more effectively identifies high-risk patients requiring active treatment (60). Precision technologies such as robot-assisted techniques can enable planned saturation sampling, thereby improving pathological consistency (61).

The essence of an individualized strategy is “risk-based supplemental sampling.” The “targeted + systematic” approach is used for broad screening to ensure no cases are missed; the “targeted + saturation” approach is used for in-depth investigation of key areas to avoid underestimating risk. Clinical selection depends on a precise balancing of the risks of missed diagnosis and overdiagnosis for each patient.

The key to safely implementing a unilateral lobe biopsy strategy lies in establishing quantifiable, low-risk screening criteria. Recent studies have provided clear evidence-based boundaries for the clinical application of this strategy.

Although for clear unilateral imaging lesions, contralateral systematic biopsy detects only about 3% more csPCa, these missed tumors are predominantly high-risk types; therefore, indiscriminately omitting contralateral biopsy carries risks (62). The key to safe implementation lies in combined screening: when patients meet the criteria of a “clear unilateral lesion” and “low PSAD”, the risk of occult csPCa in the contralateral lobe can be reduced to an extremely low level (<1%) (63). In such strictly selected patients, treatment plans based on unilateral targeted biopsy are appropriate and reliable, and there is no substantial difference in treatment outcomes compared to combined biopsy approaches (64).

Unilateral lobar biopsy is not a universal strategy, but rather an individualized approach based on dual screening using strict imaging and clinical biochemical indicators. For eligible patients, this strategy safely enables minimally invasive diagnosis without compromising subsequent critical treatment decisions

## 5 Clinical validation of the new paradigm: research progress and practical pathways for biopsy-free diagnosis

With the rapid advancement of mpMRI, PSMA-PET/CT, and risk stratification models, PCa diagnosis is gradually moving toward a new phase of “biopsy-free” diagnosis. This paradigm aims to directly guide treatment decisions through high-precision imaging and biomarkers, and is particularly suitable for preoperative assessment and intraoperative rapid pathological verification. This chapter systematically reviews the clinical validation evidence for biopsy-free diagnosis in decision-making for radical prostatectomy(RP), the auxiliary role of intraoperative frozen section biopsy, and key points for establishing practical pathways.

### 5.1 Preoperative biopsy-free diagnosis of PCa: evidence and selection criteria

The prerequisite for RP based on biopsy-free diagnosis is that imaging and clinical indicators strongly suggest csPCa, with an extremely low risk of misdiagnosis. Multiple studies in recent years have provided evidence-based support for this approach.

High consistency between imaging and clinical markers serves as a reliable foundation for implementing a biopsy-free strategy. Studies indicate that combining the PI-RADS score with PSAD can effectively screen high-risk patients; when PI-RADS is 4–5 and PSAD >0.15 ng/mL/cc, the positive predictive value for csPCa exceeds 90% (65). Further zone-specific analysis indicates that patients with a PI-RADS 5 score in the peripheral zone and a PSAD >0.20 ng/mL/cc are at extremely high risk, which can directly inform treatment decisions and obviate the need for preoperative biopsy (66). Furthermore, mpMRI-based radiomics models have demonstrated high diagnostic performance in predicting high-grade cancer (67), further corroborating the deep association between imaging features and tumor aggressiveness. Collectively, this evidence supports the feasibility of safely simplifying diagnostic pathways by leveraging the consistency between imaging and biomarkers.

The clinical feasibility of biopsy-free surgery has been validated by prospective studies. This is based on the establishment of precise imaging pathways: the combination of mpMRI and targeted biopsy significantly optimizes diagnostic performance (68, 69), while dual-modality assessment using mpMRI and PSMA-PET/CT can accurately identify very high-risk patients (70). Based on this, confirmatory studies on direct surgery have shown that among patients selected using strict imaging criteria (e.g., PI-RADS ≥ 4 and PSMA-PET positivity), the proportion of pathologically confirmed high-grade cancer following biopsy-free RP is extremely high (up to 96.7%), with no major misdiagnoses (71, 72). A recent prospective single-center study conducted by Wang et al. further solidified this evidence (73). This study employed stricter inclusion criteria (e.g., PI-RADS score ≥4, maximum standard uptake value on PSMA-PET/CT ≥6.5,PSAD ≥ 0.20 ng/mL/cc) and performed biopsy-free RP in 49 highly selected patients. Postoperative pathology confirmed a 100% rate of csPCa, with no cases of indolent cancer (Gleason score ≤ 6) being misdiagnosed. “These results not only reaffirm the feasibility of the biopsy-free diagnosis but also elevate the consistency between preoperative diagnosis and postoperative pathology to a new level through the application of stricter screening thresholds.”

Although the aforementioned prospective studies all support the implementation of biopsy-free RP under strict screening criteria, there are differences among the studies regarding specific inclusion criteria (e.g., PSAD threshold, PI-RADS score, definition of PSMA-PET/CT positivity) and exclusion criteria (e.g., history of prior treatment, evidence of metastasis). To facilitate comparison of how these differences affect the generalizability of the study conclusions, we summarize them as follows (**Table 2**).

**Table 2:** Comparison of inclusion/exclusion criteria in key prospective studies.

Study title (First author, year)	Study design	Key inclusion criteria	Key exclusion criteria	Primary outcome
Kasivisvanathan (PRECISION, 2018) (1)	RCT	Elevated PSA (≤20 ng/mL), biopsy-confirmed, treatment-naive	Contraindications for mpMRI, previously positive biopsy	csPCa detection rate
Bryant (TRANSLATE, 2025) (20)	RCT	Suspected PCa requiring initial or repeat biopsy	History of antibiotic allergy, coagulation disorders	csPCa detection rate, incidence of sepsis
Niu (2024) (71)	Prospective single-arm	PI-RADS 4–5, PSMA-PET-positive, PSAD ≥ 0.20	History of PCa treatment, evidence of metastasis	Postoperative csPCa pathological concordance rate
Wang (2025) (73)	Prospective single-arm	PI-RADS 4–5, PSMA-PET SUVmax ≥ 6.5, PSAD ≥ 0.20	Same as Niu, plus MRI-PET image mismatch	Postoperative csPCa pathological concordance rate

A comparison reveals that as screening criteria become stricter (from qualitative PSMA positivity in the Meissner study, to PSAD ≥ 0.20 in the Niu study, and further to the requirement of SUVmax ≥ 6.5 in the Wang study), the concordance between postoperative pathology and preoperative diagnosis shows an upward trend. This suggests that in future clinical practice, centers should select appropriate thresholds based on their own imaging quality and maintain a cautious approach even under more lenient standards.

A patient selection and shared decision-making model is a key safeguard for the safe implementation of biopsy-free diagnosis. The development of this model begins with clear, evidence-based selection criteria. For example, in its 2025 guideline update, the French Association of Urology has begun to systematically integrate indicators such as mpMRI and PSAD, providing an initial official recommendation framework for identifying high-risk patients who may be suitable for deferral of immediate biopsy (74). Building on these standards, the core of the decision-making process lies in shared risk communication and individualized choices between physician and patient. As in the AS pathway, physicians must use clear risk stratification tools to fully explain to patients the benefits and uncertainties of different options—including biopsy-free treatment—ultimately reaching an individualized decision based on shared values (75). Therefore, a mature biopsy-free pathway model essentially combines the standard consensus of guidelines with the established concept of shared decision-making in AS to form a structured clinical practice framework.

However, the widespread clinical application of “biopsy-free diagnostic strategy” still faces practical challenges. Their success relies heavily on high-quality, standardized image acquisition and interpretation, which is difficult to guarantee in regions with uneven distribution of medical resources. Furthermore, the long-term oncological safety of this strategy requires validation through additional prospective, large-scale data. Therefore, current practice should be strictly limited to the framework of experienced multidisciplinary teams, and should be implemented cautiously in highly selected patients following full informed consent.

### 5.2 The role of intraoperative needle biopsy: real-time navigation for precision surgery

For patients undergoing radical surgery, intraoperative frozen section biopsy can serve as a supplementary or confirmatory tool for biopsy-free diagnosis, offering real-time navigational value particularly in decisions regarding neurovascular bundle preservation or extended lymph node dissection.

The core role of intraoperative needle biopsy (rapid frozen section pathology) lies in providing real-time navigation for RP to achieve an optimal balance between tumor control and functional preservation. A prime example of its application is the technique of frozen section examination adjacent to neurovascular bundles. A 20-year retrospective analysis confirms that the real-time intraoperative application of this technique can safely increase the nerve-sparing rate by more than 20% without increasing the risk of positive margins, significantly improving patients’ functional outcomes (76). A recent systematic review and meta-analysis further provides high-level evidence-based support for this method, confirming its effectiveness in reducing the rate of positive margins and establishing it as a key tool for enhancing surgical precision (77). With advances in surgical techniques, real-time pathological navigation—represented by intraoperative frozen section analysis—has become an indispensable component of new-generation precision surgeries, such as robot-assisted procedures. By providing immediate feedback to guide surgeons in making individualized adjustments to the resection margin, it represents the future direction of precision surgical technology (78).

Intraoperative lymph node assessment is evolving toward a precision paradigm that combines “molecular imaging-guided precision targeting” with “real-time pathological safety verification.”

Molecular imaging navigation has enabled a shift from “blind scanning” to visualization. Studies have confirmed that this technology is not only feasible but also provides clear treatment-free survival benefits for patients with positive lymph nodes, establishing a new standard for functional navigation (79, 80). Intraoperative frozen section pathology serves as a critical safety net. Meta-analyses have shown that NeuroSAFE significantly improves nerve preservation rates and reduces positive surgical margins (77, 81). This finding has been further validated by the phase 3 NeuroSAFE PROOF trial, which demonstrated that NeuroSAFE-guided robot-assisted RP significantly improved 12-month erectile function and early urinary continence compared with standard surgery, without compromising oncological safety (82).

Through real-time histological verification, it ensures maximum functional preservation while guaranteeing tumor radical resection.

The feasibility of integrating intraoperative frozen section biopsy with biopsy-free diagnosis stems primarily from the precise screening of csPCa using advanced imaging. Recent prospective studies have confirmed that for patients screened based on strict imaging criteria, biopsy-free RP is safe and effective, with a very high proportion (up to 96.7%) of postoperative pathological confirmation of high-grade cancer (71, 72).

Building on this, intraoperative frozen section technology serves as a critical intraoperative verification tool that can further optimize surgical decision-making and outcomes. A large-scale meta-analysis demonstrated that integrating NeuroSAFE into robot-assisted radical resection significantly increases the rate of nerve preservation (OR 5.49) and reduces the rate of positive margins (OR 0.55) (82). Through standardized real-time pathological assessment, this technology guides immediate secondary resection upon detection of positive margins, thereby safely expanding the indications for functional preservation without compromising tumor radicality (83).

Ultimately, this integrated strategy translates into improved patient outcomes. Studies show that patients undergoing NeuroSAFE-guided surgery have significantly higher rates of postoperative complete urinary continence and restoration of erectile function (82, 84). Therefore, the biopsy-free diagnosis is not merely a decision to omit biopsy, but rather a precision surgical pathway that maximizes both oncological safety and functional preservation through the core component of intraoperative real-time pathological verification.

### 5.3 Comprehensive clinical pathway for biopsy-free diagnosis: end-to-end management from imaging to treatment

**Figure 1:**
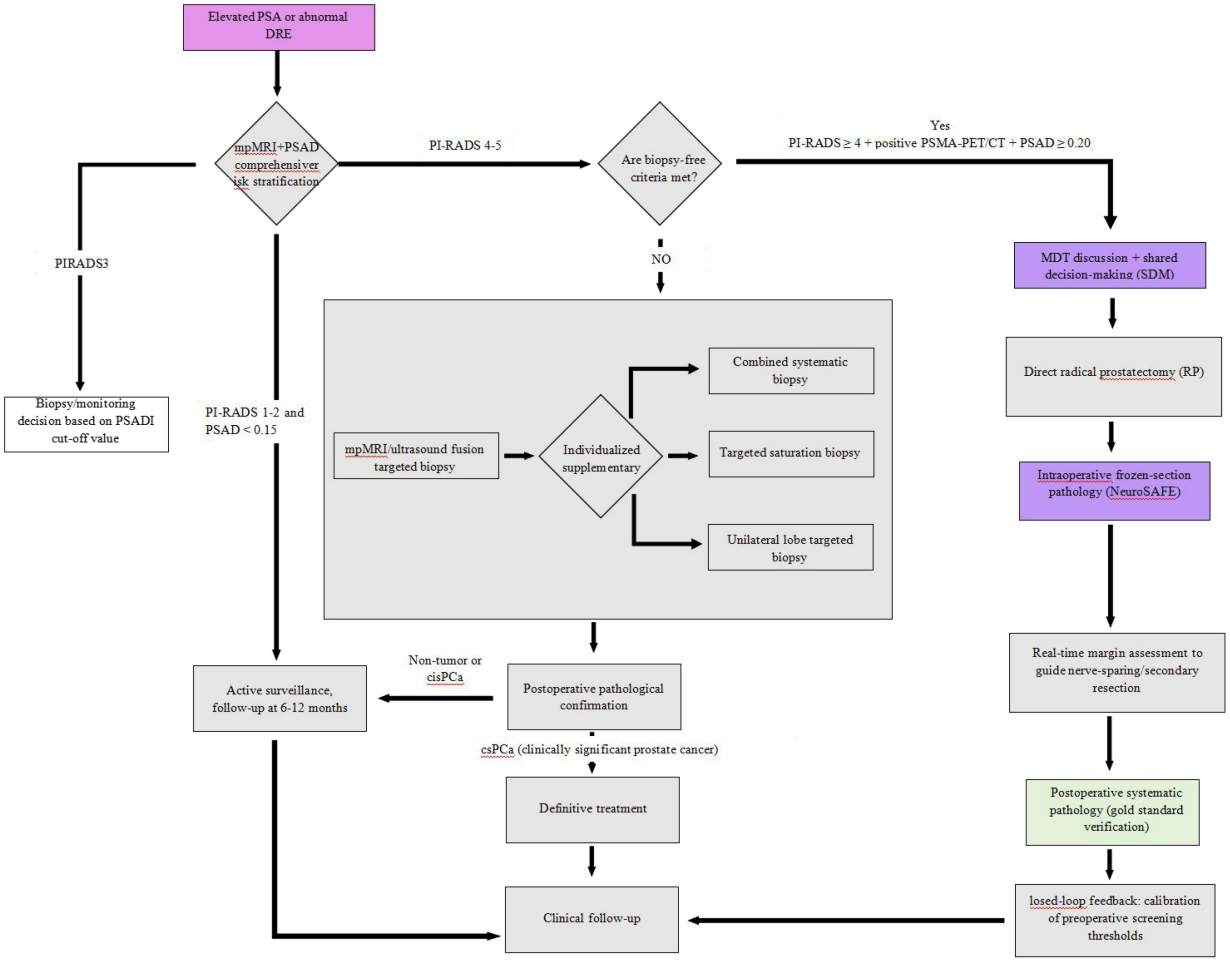
Structured clinical pathway for PCa diagnosis. This pathway integrates mpMRI/PSAD-based risk stratification, transperineal targeted biopsy, a biopsy-free diagnosis under strict criteria (PI-RADS ≥ 4 + PSMA-PET positive + PSAD ≥ 0.20), and intraoperative frozen section verification, forming a closed-loop management process from initial screening to postoperative feedback. Solid arrows in the figure represent the main pathway, while dashed lines indicate feedback or alternative pathways. MDT, multidisciplinary team.

The implementation of biopsy-free diagnosis requires the establishment of a standardized pathway covering patient selection, imaging quality control, multidisciplinary decision-making, and a closed-loop postoperative verification system. This proposed pathway is illustrated in **Figure 1**.

Establishing a screening process based on imaging and biomarkers is central to the modern paradigm of precision diagnosis for PCa, aiming to achieve efficient patient stratification and individualized management through the integration of multimodal information. The construction of this process relies on the comprehensive integration of basic imaging with advanced molecular imaging, traditional biomarkers with novel biomarkers, and visual assessment with intelligent algorithms.

First, mpMRI serves as the cornerstone of this process. Its PI-RADS scoring system provides a standardized risk stratification framework that significantly optimizes the biopsy decision-making pathway and has established a pivotal role throughout the entire process from screening and diagnosis to treatment (85, 86). Building upon this imaging foundation, PSAD serves as a critical risk “amplifier” and “refiner”. Studies have shown that in lesions with PI-RADS ≥ 3, PSAD can further accurately distinguish the risk of csPCa, particularly in “gray-zone” lesions such as PI-RADS 3, effectively improving screening specificity (87). This combined “mpMRI+PSAD” approach has been validated as an efficient diagnostic strategy superior to non-risk-stratified pathways (88).

To further improve screening accuracy for challenging cases, the workflow can incorporate more advanced imaging and molecular tools. PSMA-PET/CT can more precisely guide targeted biopsy and staging in high-risk patients, providing critical information especially when conventional imaging is inconclusive (89). Concurrently, novel blood biomarkers (such as Proclarix) can provide additional molecular biological evidence for challenging biopsy decisions, thereby optimizing patient selection (90).

To systematize these multifaceted indicators, the development of these processes is moving toward automation and intelligence. Fully automated mpMRI analysis models based on deep learning can objectively assess cancer risk. Nomograms constructed by combining these models with clinical data such as PSAD and age demonstrate significant potential for avoiding unnecessary biopsies, with performance comparable to or even surpassing that of traditional PI-RADS assessments (91). Furthermore, efforts to optimize existing PI-RADS scoring rules (such as upgrade criteria) are ongoing, aiming to further balance the trade-off between cancer detection and biopsy avoidance (92). This dynamic, multi-tiered screening workflow is also applicable to specific clinical scenarios, such as monitoring for recurrence following high-intensity focused ultrasound therapy, where specific biochemical and imaging criteria must be integrated to determine whether to perform a biopsy (93).

In summary, an ideal screening process is not the application of a single technology, but rather a dynamic system that uses mpMRI as the primary screening framework, clinical biomarkers such as PSAD as risk calibrators, and incorporates advanced tools such as PSMA-PET/CT and liquid biopsy as needed, ultimately achieving quantitative and individualized decision-making through algorithmic models. It represents a fundamental shift from a “one-size-fits-all” biopsy strategy to a precision medicine model based on continuous risk assessment.

The ultimate validation of biopsy-free diagnosis relies on the “gold standard” of post-RP pathology. Postoperative pathology not only confirms the diagnosis but also serves as critical quality feedback for optimizing the preceding non-invasive screening process.

Core evidence shows that among patients who underwent direct surgery following screening based on strict multimodal imaging criteria, postoperative pathology confirmed the presence of csPCa in 96.7% of cases, with no major misdiagnoses (73). These results are highly consistent with similar prospective studies (71, 72), collectively confirming the high reliability of current precision screening criteria. More importantly, the correlation analysis between postoperative pathology and preoperative non-invasive indicators (such as PI-RADS scores, PSAD, and PSMA-PET/CT SUV values) forms a closed-loop system for continuous quality improvement. By analyzing a small number of cases where pathology does not fully align with preoperative assessments, screening thresholds can be continuously calibrated—for example, by refining PI-RADS subclassifications, optimizing PSAD cutoffs, or exploring the value of integrating novel biomarkers—thereby ensuring oncological safety while expanding the indications for biopsy-free diagnosis.

Postoperative pathology serves as an indispensable “safety valve” and “calibrator” for the biopsy-free diagnosis. It not only validates the effectiveness of the current precision screening pathway but also drives its continuous optimization through feedback loops, serving as the cornerstone for the safe and individualized implementation of this strategy.

## 6 Discussion and outlook

The diagnosis of PCa is undergoing a profound transformation, shifting from “empirical blind biopsy” to “image-guided precision,” and ultimately toward “information-driven non-invasive” approaches. Currently, a modern diagnostic system centered on mpMRI as the primary screening “gatekeeper,” transperineal biopsy as the standard approach, and targeted biopsy as the core strategy has been largely established, while the “biopsy-free diagnosis” pathway based on rigorous multimodal screening has also demonstrated solid clinical prospects.

Looking ahead, the diagnostic paradigm will further evolve toward multidimensional integration, intelligent dynamics, and the integration of diagnosis and treatment. Artificial intelligence (AI) will deeply integrate radiomics, liquid biopsy, and clinical data to construct a full-cycle dynamic risk model spanning screening to monitoring, enabling continuous optimization and personalization of decision-making. The role of molecular imaging, exemplified by PSMA, will continue to expand. It will serve not only as an advanced navigation tool but also as a “diagnosis-treatment integration” hub connecting diagnosis and targeted therapy, enabling precise interventions based on the principle of “see and treat.” Concurrently, the prudent expansion of “biopsy-free diagnosis” strategies and the enhancement of intraoperative real-time pathological verification will jointly drive the development of safer, minimally invasive, and personalized clinical pathways.

In the future, PCa diagnosis and treatment will no longer be an isolated process ending with tissue acquisition, but will evolve into a patient-centered, real-time, multi-modal data-driven, and AI-assisted decision-making ecosystem for comprehensive health management. Through continuous technological innovation and rigorous clinical validation, we aim to ensure oncological safety while minimizing physical and psychological burdens, thereby securing better long-term quality of life and prognosis for patients.
